# Antiviral, Immunomodulatory, and Free Radical Scavenging Activities of a Protein-Enriched Fraction from the Larvae of the Housefly, *Musca domestica*

**DOI:** 10.1673/031.013.11201

**Published:** 2013-10-22

**Authors:** Hui Ai, Furong Wang, Na Zhang, Lingyao Zhang, Chaoliang Lei

**Affiliations:** 1 Hubei Key Laboratory of Genetic Regulation and Integrative Biology, College of Life Science, Central China Normal University, Wuhan 430079, P. R. China; 2 Key Laboratory of Insect Resource Utilization & Sustainable Pest Management of Hubei Province, College of Plant Science and Technology, Huazhong Agricultural University, Wuhan 430070, P. R. China; 3 Hubei Provincial Center for Diseases Control and Prevention (CDC), Hubei Provincial Academy of Preventive Medicine, Wuhan 430079, P. R. China

**Keywords:** antioxidant, immunomodulation

## Abstract

In our previous study, protein-enriched fraction (PEF) that was isolated from the larvae of the housefly, *Musca domestica* L. (Diptera: Muscidae), showed excellent hepatoprotective activity as well as the potential for clinical application in therapy for liver diseases. In this study, antiviral, immunomodulatory, and free radical scavenging activities of PEF were evaluated. The antiviral results demonstrated that PEF inhibited the infection of avian influenza virus H9N2 and had a virucidal effect against the multicapsid nucleopolyhedrovirus of the alfalfa looper, *Autographa californica* Speyer (Lepidoptera: Noctuidae) *in vitro*. The mortality of silkworm larve in a PEF treatment group decreased significantly compared with a negative control. PEF showed excellent scavenging activity for 1,1-diphenyl-2-picrylhydrazyl and superoxide anion radicals, which were similar to those of ascorbic acid. The imunomodulatory results suggested that PEF could effectively improve immune function in experimental mice. Our results indicated that PEF could possibly be used for the prophylaxis and treatment of diseases caused by avian influenza virus infection. In addition, PEF with virucidal activity against insect viruses might provide useful for the development of antimicrobial breeding technology for economically important insects. As a natural product from insects, PEF could be a potential source for the discovery of potent antioxidant and immunomodulatory agents.

## Introduction

The housefly, *Musca domestica* L. (Diptera:Muscidae), lives in filthy niches for its entire life. The larvae of the housefly and blowfly commonly are found in decaying organic matter. They spread various diseases between humans and other animals, but they can inhibit viruses or bacteria infecting them. (Liang et al. 2006). The larvae of the blowfly, *Lucilia sericata* Meigen (Calliporidae) has been used to debride and heal wounds, and to treat spleen and stomach diseases for centuries (Li 1981; Sherman et al. 1996; Prete 1997). And some antibacterial proteins have been found in larvae of the *M. domestica* (Fu et al. 2009; Dang et al. 2010). The larvae of housefly are an excellent source of high-quality protein, polyunsaturated fats, polysaccharides, vitamins, minerals and other nutrients for both human food and animal feed (Ren et al. 2002; Feng et al. 2010).

In our previous study (Wang et al. 2007), protein-enriched fraction (PEF) was isolated from the larvae of *M. domestica* and hepatoprotective effects were evaluated in rats against carbon tetrachloride (CCl_4_)-induced acute hepatic damage. Our results showed that PEF possessed excellent hepatoprotective activity and could potentially be applied in clinical therapy for liver diseases (Wang et al. 2007). In our present study, the antiviral activities of PEF against avian influenza virus H_9_N_2_ (AIV), the multicapsid nucleopolyhedrovirus (AcMNPV) of the alfalfa looper, *Autographa californica* Speyer (Lepidoptera: Noctuidae), and the nuclear polyhydrosis virus (BmNPV) of *Bombyx mori* L. (Bombicidae) were investigated. The immunomodulatory effects of PEF were evaluated in terms of body weight, phagocytic function of macrophages, 2, 4- dinitrofluorobenzene (DNFB)-induced delayed type hypersensitivity reaction, proliferation of lymphocytes, natural killer cell activity (NKCA), and hemolytic activity in mice. The free radical scavenging activity of PEF was also measured.

## Materials and Methods

### Materials

AcMNPV, BmNPV, and *Spodoptera frugiperda* cell line 9 (sf9 cell line) were provided by Dr. Xiulian Sun of the Wuhan Institute of Virology, Chinese Academy of Sciences, Hubei, P. R. China. AIV was provided by the College of Animal Medicinal Science, Huazhong Agricultural University, Hubei, P.R. China. Sf9 cells were maintained at 28° C in Grace's medium (Gibco, http://www.invitrogen.com) supplemented with 10% fetal bovine serum (Invitrogen, http://www.invitrogen.com). RPMI 1640 medium was purchased from Gibco. Medium was supplemented with 10% fetal calf serum (Gibco). 1,1-diphenyl-2-picrylhydrazyl (DPPH), Concanavalin A, lipopolysaccharide (LPS), 3-[4, 5-dimethylthiazol]-2, 5- diphenyltetrazolium bromide, and DNFB were purchased from Sigma-Aldrich (http://www.sigmaaldrich.com). All other chemicals and reagents used were of analytical reagent grade.

### Experimental animals and treatment

Specific pathogen free *Kunming* mice (18–22 g) were provided and fed at the Experimental Animal Center of Hubei Provincial Academy of Preventive Medicine, Wuhan 430079, P. R. China. Institutional approval license numbers for animal production and use are SCXK (Hubei) 2003–0005 and SCXK (Hubei) 2003–0014, respectively. They were randomly separated into four groups of ten each, placed in cages that were located in a room maintained at 22 ± 1° C with a 12 h:12 L:D cycle, and offered sterile diets and water. The mice were pre-treated with PEF suspension at 50 (lowdose), 100 (middle-dose), and 200 (high-dose) mg/kg·bw (microgram per kilogram of mouse's body weight) by forcedly feeding using a stomach tube (intragingival). The pretreatments were made at the same time for 20 days. At the same time, the mice of the control group were treated with normal saline in equal volume. The body weights of the mice were measured daily. Cervical dislocation was used in euthanasia of the mice.

### Preparation of the PEF from *M. domestica* larvae

The PEF was prepared from *M. domestica* larvae according to the method of Wang et al. (2007). Third-instar larvae were collected, washed with distilled water, frozen, and lyophilized. The lyophilized maggots were extracted with petroleum ether (bp 30∼60° C) in a Soxhlet apparatus for 50 hrs. The defatted extract was pulverized at a low temperature and treated using three sequences of cold (4° C) protein buffer (0.1 M citrate-Na_2_HPO_4_, 0.18 M NaCl dilution, pH adjusted to 7.0) for 0.5 hrs. After being centrifuged at 1800 g for 15 minutes at 4° C, the supernatant was transferred to a new container and acidified to pH 5.8 with 0.01 M HCl. The solution was fractionated at 65% saturation of (NH_4_)_2_SO_4_, and the precipitate was dialyzed against a low-ionic-strength pH 7.5 buffer. The precipitate was recovered by centrifugation and dissolved in distilled water prior to dialysis. All the dialyzed extracts were centrifuged, and the supernatant was concentrated and lyophilized. The lyophilized supernatant was PEF. It was stored at -20 °C until needed.

### Antiviral assays

#### Antiviral bioactivity analysis of PEF against AIV.

lHemagglutinin was quantified based on a method described by Nayak and Reichl (2004). Serial double dilutions of the test samples (PEF, 100 µL) were made in round-bottomed, 96-well microtiter plates containing 100 µL PBS (pH 7.4). Each sample concentration was measured five times. 100 µL of red blood chicken cells (2 × 10^7^ red blood cells/mL) were added to each cell and incubated for 60–90 minutes at room temperature. The last dilution showing complete hemagglutination was taken as the end point and was recorded as HA (dilution) or log_2_HA of 0.1 mL, respectively. Additionally, a parallel HA standard and a positive control using chicken cells in buffer were run to control the assay.

#### Antiviral bioactivity analysis of PEF against AcMNPV.

The antiviral activity of PEF against AcMNPV was determined according to the method reported by Popham et al. (2004) and Wu et al. (2003). PEF (0.5 mg/mL, 1.0 mg/mL) was combined with Ac- MNPV budded virus obtained from the supernatants of AcMNPV-infected Sf9 cells at a ratio of 1:1 (v/v), gently mixed and incubated at 28° C for 1 hr. Grace's insect medium was used as a control in the absence of PEF. Virus titres of these incubations were determined by end-point dilution assay (Slavicek et al. 2001). Sf9 cells were seeded at 1 × 10^5^ cells mL^-1^ in 96-well plates and allowed to attach for 1 hr. The cells were infected with dilutions of virus/PEF or virus/medium at dilutions of 10-1 to 10-8, and plates were incubated for 1 week at 28° C. The plate wells were then scored as being positive for virus infection if polyhedra were visible within two or more cells, or negative for viral infection, and the results were used to calculate the virus titre as the TCID_50_ according to the method of Reed and Muench (1938). Each test was performed three times. Statistical comparisons were done with SPSS software (http://www-01.ibm.com/software/analytics/spss/).

#### Antiviral bioactivity analysis of PEF against BmNPV.

The antiviral activity of PEF against BmNPV was determined according to the method reported by Li et al. (2006) and Nakazawa et al. (2004). The BmNPV occlusion bodies (OB) were diluted to 2 × 10^5^ OBs/mL by buffer (0.15mol/L NaCl, 0.1 mol/L Tris-HCl, pH7.4). The BmNPV OB was mixed with PEF, and the volume was adjusted to 1 mL. The final concentration of PEF was 0.5 mg/mL, and the OB was 1 × 10^5^ OBs/mL in this mixture solution. These solutions were incubated in a water bath of 37° C for 4 hrs. Fresh mulberry, *Morus* L. (Rosales: Moraceae) leaves (4cm × 4cm) were daubed uniformly with these mixture solutions and dried at room temperature. Then, 50 thirdinstar silkworm, *Bombyx mori* L. (Lepidoptera: Bombycidae), larvae were fed with those leaves. 1 × 10^5^ OBs/mL OB solution without PEF was taken as the control, and each test was performed three times. The larval mortality rate was investigated every 24 hrs.

### Effects PEF on body weight

The body weights of the mice in four groups were measured daily.

### Immunomodulatory activities of PEF

#### Effects on macrophage phagocytosis in mice by the carbon clearance method.

Mice were injected via the tail vein with 0.1 mL of pre-warmed colloidal carbon suspension (Indian ink), which was diluted eight times with PBS (pH 7.4) before use. Blood samples were drawn from the retro-orbital plexus at 2 and 12 minutes. 25 µl of blood was dissolved in 2 mL 0.1% sodium carbonate, then the absorbance was measured at 660 nm (Tiwari et al. 2004). The phagocytic index, *K*, was calculated by the equation *K*= (lg OD_1_-lg OD_2_) / (t_2_-t_1_), where, OD_1_ and OD_2_ depict the optical densities at times t_1_ and t_2_, respectively.

#### DNFB-induced delayed-type hypersensitivity (DTH) reaction in mice.

Mice were sensitized by placing 20 µl 0.5% 2, 4-dinitrofluorobenzene in acetone-olive oil (1:1) on the shaved abdominal skin of recipients. Five days later, 10 µl 0.2% DNFB solution was placed on the right ear. Forty-eight hours later, ear swelling was expressed as the difference between the weight of the left and right ear patches obtained from 8-mm punches (Feng et al. 2002). The punches were from random areas of the middle of the ear. Ear swelling rate was calculated according to the following equation: Ear swelling rate = [(weight of the right ear − weight of the left ear) / (weight of the left ear)] × 100%.

#### Preparation of spleen cell suspension and lymphocyte proliferation assay.

Spleens were aseptically obtained from the mice and placed in complete RPMI 1640 medium. Then, a single-cell suspension was prepared by teasing the spleens apart and filtering it through steel mesh (200 mesh). Erythrocytes were lysed with Tris-buffered ammonium chloride (0.155 M NH_4_Cl and 16.5 mM Tris, pH 7.2). The cells were washed twice and then resuspended in complete RPMI 1640 medium. The concentration of splenocytes was adjusted to 5 × l0^6^ cells/mL. Concanavalin A and lipopolysaccharide were used as mitogens, and the final concentration was 5 µg/mL. The cells were cultured for 48 hrs, and pulsed for the last 4 hrs with 10 µl per well of 3-[4, 5-dimethylthiazol]-2, 5-diphenyltetrazolium bromide (5 µg/mL). The cells were washed three times with RPMI- 1640 medium, and 200 µl dimethylsulfoxide was added into the wells of a 96-well, flatbottom, microwell plate. Each test was replicated in three wells. The absorbance (OD_570_) was measured in a microplate reader (Bio-Rad, Model 680, http://www.bio-rad.com), and the proliferation index was calculated (Ivanovska et al. 1996).

#### NKCA assay.

Lymphocytes from each mouse were prepared as above. NKCA was determined by lactate-dehydrogenase release assay based on the method described by Zhang et al. (2004). The lymphocytes were washed and suspended in complete RPMI-1640 medium, counted, and diluted to 1.0 × 10^6^/mL. The amount of the lactate-dehydrogenase released from the lysed target cells was determined for measuring NKCA. Yac-1 cell line (murine Tlymphoma cell) was used as the target cell. Yac-1 was washed with complete RPMI-1640 medium, counted, and diluted to a concentration of 1.0 × 10^5^/mL with medium. The same volume of Yac-1 cells and lymphocytes were added into the wells of a 96-well, flat-bottom, microwell plate (the cell ratio of effector-totarget was 10:1). Each test was replicated in three wells. After a 2-hr incubation at 37° C in a humid atmosphere with 5% CO_2_, the plate was centrifuged at 1000 rpm/min for 5 min. The supernatant from each well (100 µ1) was transferred into the corresponding wells of a 96-well, flat-bottom, microwell plate. Then, 100 µ1 of lactic acid dehydrogenase substrate mixture was added into each well. After 3 min, 50 µ1 cold medium was added into each well to stop reactions. Finally, the absorbances (OD_490_) were measured in a microplate reader (Bio-Rad, Model 680). The percentage of NKCA was calculated by the equation NKCA = (*E*-*S*) / (*M*-*S*) × 100%, where *E* represents the experimental release of lactatedehydrogenase activity from target cells incubated in the presence of lymphocytes, *M* represents the maximum release of the lactatedehydrogenase lactatedehydrogenase activity determined by lysing the target cells with 1% of NP-40, and *S* is the spontaneous release of the lactatedehydrogenase activity from target cells incubated in the absence of lymphocytes.

#### Hemolytic activity assay.

Hemolytic activity was determined based on the method described by Zhao et al. (2005). The test mice were immunized with sheep red blood cells on Day 0, and PEF was orally administered at the designed experimental dose from Day 0 to Day 5. On Day 6, the blood was obtained from each mouse, and the serum samples were obtained from clotted samples and diluted by saline up to 500 times. 1 mL of the serum was placed in a microcentrifuge tube. Subsequently, 0.5 mL of diluted sheep red blood cells and 1 mL of diluted guinea pig complement were added to the above tube. The tube was incubated in a water bath at 37° C for 10 minutes with occasional shaking to allow the lysis of target cells, and was followed by centrifugation. Next, 1 mL of supernatant was taken out and diluted with 3 mL distilled water and placed in a cuvette. The amount of hemoglobin released was measured by OD at 540 nm. To measure the control, the normal saline, instead of the serum, was added to the tube containing the complement and the target cells, indicating spontaneous and complement- mediated lysis of the target cells in the absence of hemoglobin. The complete control hemolysis was achieved by incubating the processed serum with 1% Tween 20 in saline. The hemolytic activity was calculated as percentage according to the formula: Hemolytic activity = [(mean A540 nm value of the serum samples - mean A540 nm value of the saline samples) / (mean A540 nm value of the Tween 20 samples - mean A540 nm value of the saline samples)] × 100%.

Serum samples from all the mice were obtained from clotted samples and serially diluted (1:80) in normal saline. Serum of mice 0.5 mL and 5% sheep red blood cells 0.5 mL were mixed with serum of guinea pig (1:5) 0.5 mL and normal saline 0.5 mL. HC_50_ was calculated by A_540_.

### Scavenging ability on DPPH radical

The scavenging effect of PEF on 1,1-diphenyl-2-picrylhydrazyl radical was studied, employing the modified method described earlier by Ai et al. (2008b). Briefly, 400 µMDPPH solution in methanol was prepared, and 3.0 mL of this solution was added to 1.0 mL test samples at different concentrations (0.0625 mg/mL, 0.125 mg/mL, 0.25 mg/mL, 0.5 mg/mL, 1.0 mg/mL, 2.0 mg/mL, 4.0 mg/mL). The reaction mixture was shaken well and incubated for 30 min at room temperature, and the absorbance of the resulting solution was read at 517 nm against a blank. IC_50_ value is the effective concentration at which DPPH radicals were scavenged by 50% and was obtained by interpolation from linear regression analysis. Ascorbic acid was used for comparison. Each test was replicated three times. The radical scavenging ability was calculated using the following equation:

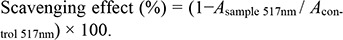



### Superoxide anion radical scavenging assay

The superoxide scavenging ability of sulfated PEF was assessed by the method of Xing et al. (2005). The reaction mixture, containing PEF (0.125, 0.25, 0.5, 1.0, 2.0 mg/mL), phenazine methosulfate (30 µM), nicotinamide adenine dinucleotide (338 µM), and nitro blue tetrazolium (72 µM) in phosphate buffer (0.1 M pH 7.4) was incubated at room temperature for 5 min and the absorbance was read at 560 nm against a blank. Ascorbic acid was used for comparison. Each test was replicated three times. The capability of scavenging to superoxide radical was calculated using the following equation:

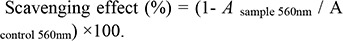



### Statistical analysis

Using SPSS, the significance of the differences between PEF treated groups and the control group was evaluated by Student's t-test at *p* < 0.05 and *p* < 0.01.

## Results

### The antiviral bioactivity of PEF against AIV

**Figure 1.**
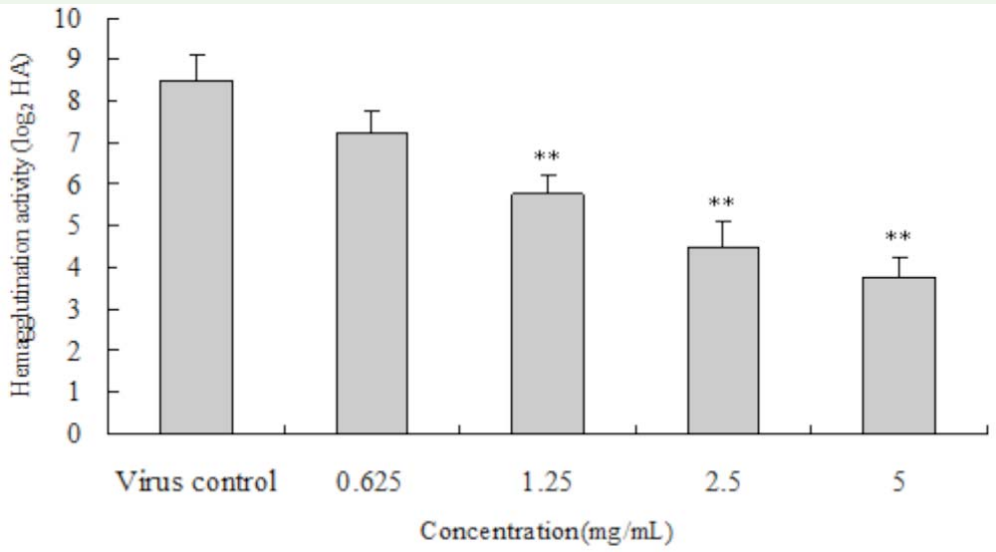
The antiviral effects of protein-enriched fraction (PEF) from *Musca domestica* larvae against avian influenza virus. The significance of the difference between the virus control and the treated group was determined by Student's *t*-test. Each test was replicated three times. **p < 0.01. High quality figures are available online.

Avian influenza virus H_9_N_2_ adsorbs to chicken red blood cells, resulting in hemagglutination. We investigated if PEF could interfere with the AIV adsorption to red blood cells that would result in inhibition of hemagglutiniation. As shown in Figure 1, PEF exhibited significant antiviral activity in the concentration range of 1.25mg/mL, 2.5 mg/mL, and 5.0 mg/mL.

### The antiviral bioactivity of PEF against AcMNPV

**Figure 2.**
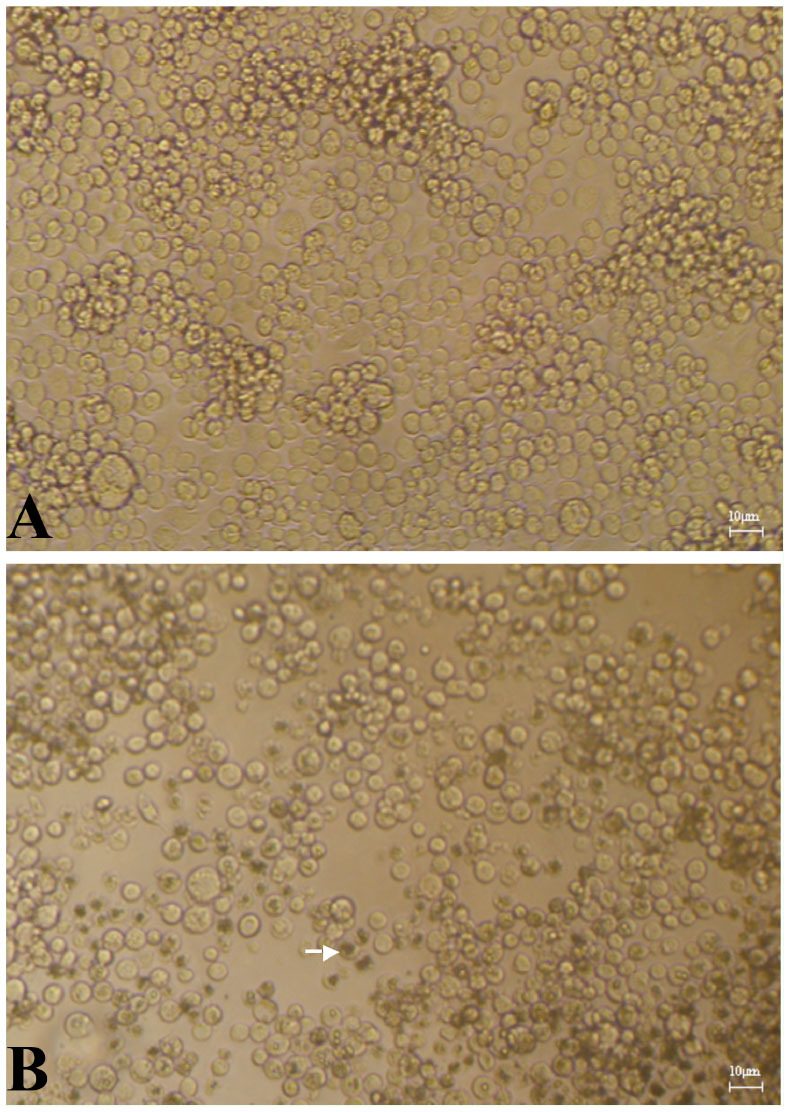
Photomicrographs of normal *Spodoptera frugiperda* 9 cells (A) and pathological *Spodoptera frugiperda* 9 cells (B) infected by *Autographa californica* multicapsid nucleopolyhedrovirus. High quality figures are available online.

Photomicrographs of normal and sf9 cells infected with *A. californica* multicapsid nucleopolyhedrovirus are shown in Figure 2. An obvious cytopathic effect of PEF on sf9 cells in the viral infection group was observed after 72 hrs. AcMNPV was released from infected cells, and a large number of nuclear polyhedra were observed within cell medium (Figure 2B). Normal sf9 cells had better cell growth, and a cytopathic effect was not observed in the control group (Figure 2A).

### The effect of PDF on virus titer assayed on sf9 cells

**Figure 3.**
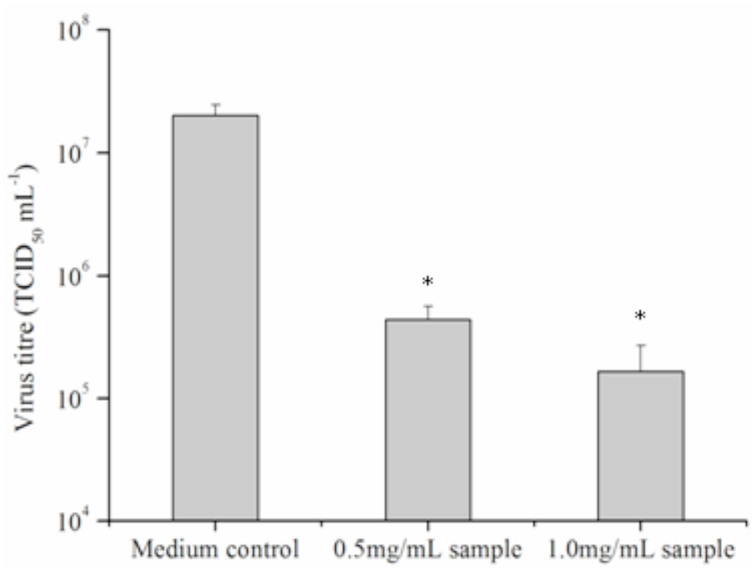
The antiviral effects of protein-enriched fraction from *Musca domestica* larvae against *Autographa californica* multicapsid nucleopolyhedrovirus *in vitro*. The significance of the difference between the medium control and the treated group was determined by Student's *t*-test. Each test was replicated three times. *p < 0.05. High quality figures are available online.

The antiviral activity of PEF from the larvae of *M. domestica* was detected using a TCID_50_ assay for virus titer. Incubation of PEF (1.0 and 0.5mg/mL) with AcMNPV *in vitro* at 20° C for 1 hr dramatically reduced the TCID_50_ mL^-1^ when dilutions were subsequently assayed on sf9 cells. The virus titre declined significantly from 2.0 × 10^7^ ± 0.42 × 10^7^ to 4.4 × 10^5^ ± 1.2 × 10^5^ (0.5mg/mL PEF) and 1.7 × 10^5^ ± 2.1 × 10^4^ (1.0mg/mL PEF) TCID_50_ mL^-1^ (*p* < 0.05), indicating the PEF from the larvae showed significant virucidal activity against AcMNPV (Figure 3).

### The antiviral bioactivity of PEF against BmNPV

**Figure 4.**
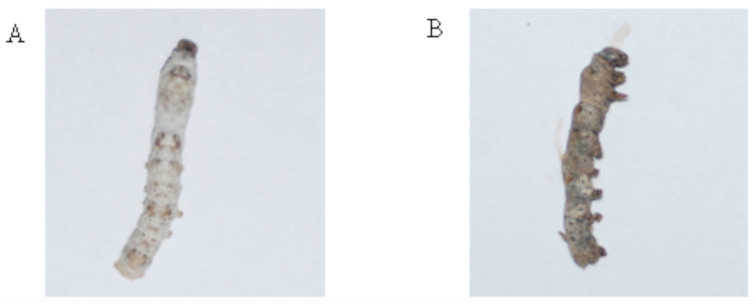
Photomicrographs of normal *Bombyx mori* larvae (A) and pathological *B. mori* larvae (B) infected by *B. mori* nuclear polyhydrosis virus. High quality figures are available online.

**Figure 5.**
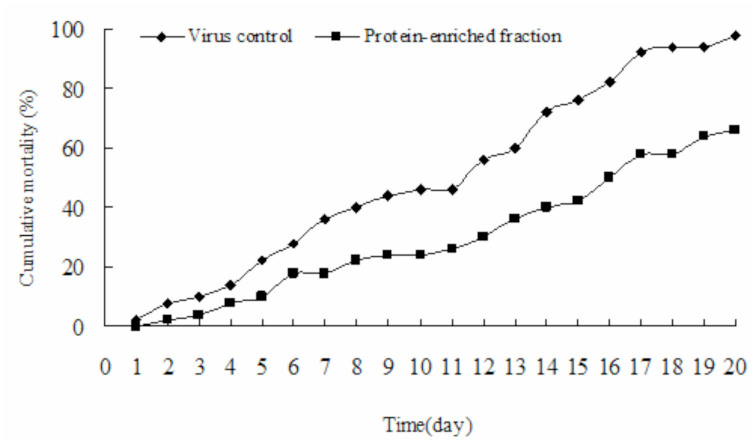
The time-mortality relationship of *Bombyx mori* after infection of *B. mori* nuclear polyhydrosis virus. Each test was replicated three times. High quality figures are available online.

After infection of *B. mori* larvae with nuclear Bm polyhydrosis, pathological changes were observed in the infected group (Figure 4B). As shown in Figure 5, after treatment of BmNPV occlusion bodies *in vitro* with PEF (0.5 mg/mL) and injection into *B. mori*, it was found that larval morality decreased significantly. This result demonstrated that PEF could effectively inhibit the infectivity of the BmNPV occlusion bodies.

### Immunomodulatory activities of PEF

#### Effects on body weight

At the last day of the treatment with PEF, the body weights of the mice were measured. The result showed that body weight was significantly increased (*p* <0.05) only in the mice receiving the high dose of PEF and the effect was marginal (Table 1).

#### Effects on macrophage phagocytosis using the carbon clearance method

The phagocytic index of all the treated groups increased significantly compared with the control group (*p* < 0.05, Table 1).

**Table 1.**
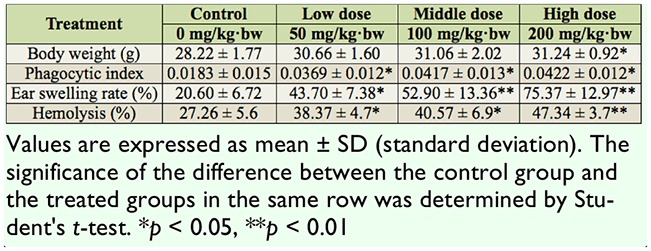
The effect of protein-enriched fraction (PEF) isolated from the larvae of housefly on body weight, phagocytic activity, 2, 4-dinitrofluorobenzene-induced ear swelling, and hemolytic activity in mice.

#### Effect of PEF on ear swelling DNFBinduced DTH response

DNFB-induced DTH reaction is a Th1 cellmediated pathologic response involved with T cell activation and the production of many cytokines. The effects of PEF on DTH responses in mice are illustrated in Table 1. Compared to the control group, all doses significantly augmented ear swelling (*p* < 0.05 and p <0.01).

#### Effect of PEF on the lymphocyte proliferation

**Figure 6.**
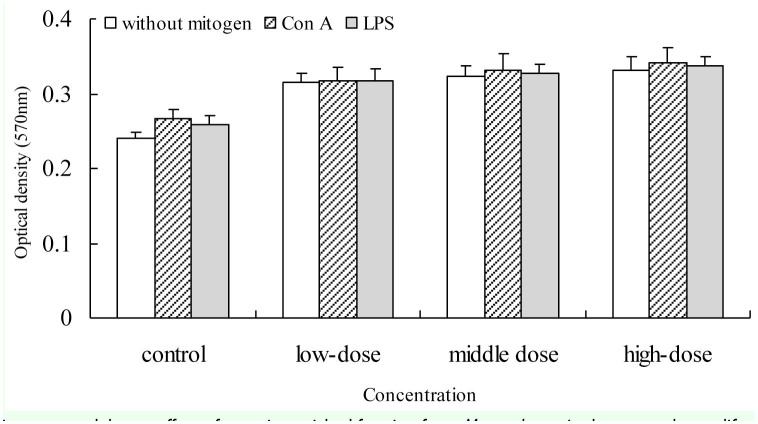
The immunomodulatory effect of protein-enriched fraction from *Musca domestica* larvae on the proliferation of splenic lymphocytes in mice. The proliferation of splenic lymphocyte was measured by the MTT method. The significance of the difference between the control group and the treated groups was determined by Student's *t*-test. *p < 0.05. High quality figures are available online.

In order to confirm the effect of PEF on the cellular immune response, the proliferation of splenocytes from mice in response to concanavalin A and LPS was evaluated. The results indicated that PEF can increased the proliferation of spleen cells compared to the control group (Figure 6).

#### NKCA

**Figure 7.**
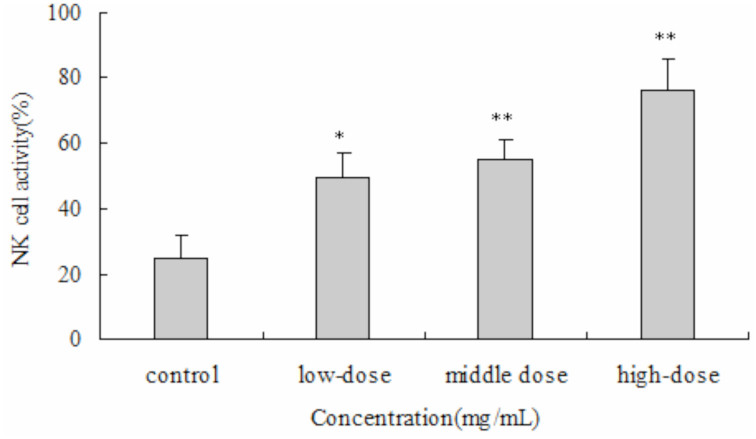
The immunomodulatory effect of protein-enriched fraction from *Musca domestica* larvae on the natural killer cell activity in mice. Natural killer cell activity was determined by lactate dehydrogenase release assay. The significance of the difference between the control group and the treated groups was determined by Student's *t*-test. *p < 0.05, **p < 0.01. High quality figures are available online.

The results showed that the natural killer cell activity of the all doses of PEF significantly increased killer cell activity (*p* <0.05 and *p* 0.01) . Moreover, the effect of PEF was dosedependant (Figure 7).

#### Hemolytic activity

At the dosages of 50, 100, and 200 mg/kg·bw, significant increases in a dose-dependant manner (*p* <0.05 and *p* <0.01) were observed in the hemolytic activity of PEF treated groups compared with the control group (Table 1).

#### DPPH radical scavenging activity

**Figure 8.**
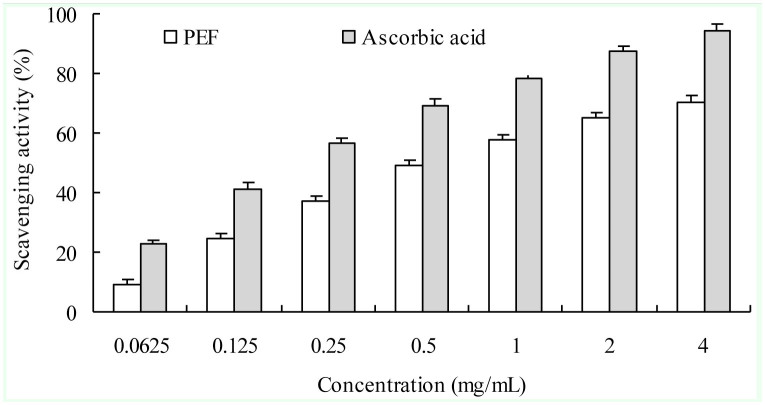
1,1-diphenyl-2-picrylhydrazyl radical scavenging effects of protein-enriched fraction from *Musca domestica* larvae and ascorbic acid. Each test was replicated three times. The bar represents +SD. High quality figures are available online.

As shown in Figure 8, the scavenging effect of PEF on 1,1-diphenyl-2-picrylhydrazyl radical exhibited a concentration-dependent manner, but its value remained lower than ascorbic acid. The IC_50_ of PEF and ascorbic acid was approximately 0.72 mg/mL and 0.22 mg/mL, respectively.

#### Superoxide anion radical scavenging activity

**Figure 9.**
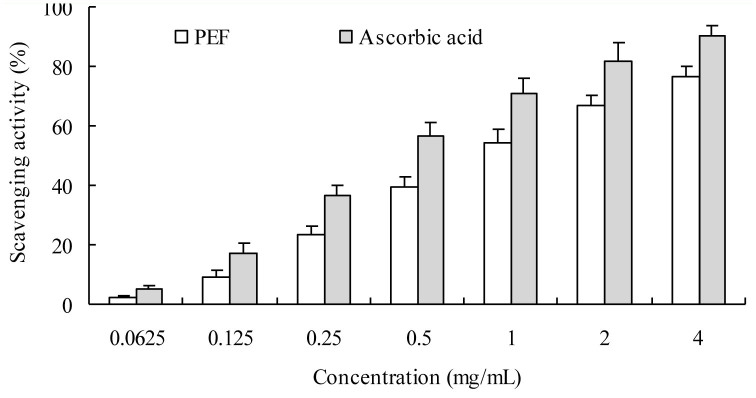
Superoxide anion radical scavenging effects of protein-enriched fraction from *Musca domestica* larvae and ascorbic acid. Each test was replicated three times. The bar represents +SD. High quality figures are available online.

The inhibitory effect of the PEF on superoxide anion radical showed high scavenging activity for superoxide radical, which was similar to that of ascorbic acid (Figure 9). The IC_50_ of PEF and ascorbic acid was approximately 0.90 and 0.47 mg/mL, respectively.

## Discussion

Insects are a large, unexplored, and unexploited source of potentially useful compounds for modern medicine (Pemberton 1999). Various biological and medicinal activities of extracts from *M. domestica* larvae have been reported, e.g., antibacterial, antiviral, anti-allergic, antiinflammatory, antitumor, and radical scavenging activity (Meylaers et al. 2004; Jin et al. 2005; Liu et al. 2011; Ratcliffea et al. 2011). Our previous experiments also suggested that the homogenate of the larvae of this fly possessed antiviral activity against HbsAg and influenza viruses and hydroxyl radical scavenging activity *in vi*tro (Wang et al. 2006). On this basis, the PEF was obtained from *M. domestica* larvae, and anviviral, immunomodulatory, and free radical scavenging activities were evaluated in this study.

Most economically important insects are susceptible to diseases caused by viruses. For example, viruses cause 70% of the damage in sericulture . Among viruses, nuclear polyhedrosis viruses, especially BmNPV have caused the most damage to silk production in recent years (Yao et al. 2008). Therefore, the investigation of antiviral active substances from natural insect resources will greatly enhance antiviral competence and genetic improvement of economic insects. Many proteins or peptides with antiviral activity against insect viruses and influenza viruses were found in tobacco budworm, *B. mori*, *Heliothis virescens*, and *Calliphora vicina* (Chernysh et al. 2002; Popham et al. 2004; Nakazawa et al. 2004; Shelby and Popham 2006). In our present study, PEF was isolated from *M.domestica* larvae and exhibited high antiviral bioactivity against BmNPV and AcMNPV, at the low concentration of 0.5mg/mL. This result suggests it could effectively inhibit the infection of BmNPV and AcMNPV. These results may help to better understand the insect immune mechanisms against viral pathogens.

There is evidence that antiviral activities are important immune functions in animals (Pierson et al. 2000; Maurizio 2005). In this study, the effects of PEF on modulation of immune function was investigated in mice in terms of phagocytic activity, DNFB induced DTH reaction, proliferation of lymphocytes, NKCA, and hemolytic activity. One of the most important nonspecific immune responses of the body, phagocytic function, is carried out by macrophages. Phagocytosis represents an important innate defense mechanism against ingested particulates, including whole pathogenic microorganisms. Our results indicated that PEF could enhance the phagocytic function of macrophages in a dose-dependant manner. The beneficial effect of PEF could be partially attributed to enhancing the effect on nonspecific immune responses of the body.

The specific immune response includes humoral immunity and cellular immunity. The immunoregulatory effect of PEF on T cell functions was confirmed in a murine DTH model. Ear swelling in DTH is primarily the result of *in vivo* functions of antigen-specific CD^4+^ T cell response (Grabbe and Schwarz 1998). The results indicated that PEF treatments significantly enhanced DTH reaction dose-dependently, which was reflected in the increased ear-swelling rate compared to the control group. Furthermore, compared with control group, PEF treatments also significantly enhanced hemolytic activity in a dosedependent manner. Together, these results suggest that PEF has the ability to modulate humoral immunity.

Lymphocyte stimulation with mitogens is a measure of cell-mediated immunity. Concanavalin A is a well-known activator of T lymphocytes, which involves the interleukin-2 (IL-2)/IL-2 receptor (IL-2R) complex. In the present study, augmentation of Concanavalin A-induced lymphocyte proliferation by PEF suggested that PEF enhanced cellular immunity (T cell function). In addition, lipopolysaccharide is an activator of T-celldependent antibody production by B cells (Whitehurst and Geppert 1996), and the augmentation of LPS-induced lymphocyte proliferation would imply that PEF also influences B cells in mice. This study demonstrated a significant increase in the proliferation of lymphocyte induced by Concanavalin A and LPS. PEF might contain active components associated with T and B cell proliferation stimulation.

It is well known that NK cells play an important role in regulating immune responses. They are important contributors to innate defenses against viral infections. NK cells could rapidly recognize and lyse a large variety of virus-infected cells, without the need for either prior sensitization or MHC-dependent recognition, which is different from cytotoxic T cell (Kodama et al. 2002). In this study, PEF enhanced the NKCA and showed a dosedependent relationship. This result suggested that PEF might possess the ability to activate NK cells to improve the immune function of body. In our previous study, PEF showed excellent hepatoprotective activity, and its hepatoprotective potential was closely related to immunomodulatory results (Wang et al. 2007).

In addition, free radical scavenging activity of PEF had been also measured in this study. The present results demonstrated that PEF possessed DPPH radical and superoxide anion radical scavenging activity. Our previous study showed that PEF not only significantly improved superoxide dismutase (SOD) and glutathione peroxidase (GSH-Px) activities of serum and liver homogenate in aged mice, but also depressed malondialdehyde (MDA) production in mice liver homogenate by autooxidation and hepatic mitochondria expanded induced by Fe^2+^-ascorbic acid system (Ai et al. 2008a). Superoxide dismutases (SOD) and glutathione peroxidase (GSH-Px) are enzymes that are an important antioxidant defense in nearly all cells exposed to oxygen. They played a crucial biological role in avoiding oxidative damage of the organism. Malondialdehyde (MDA) is a reactive oxygen species (ROS), and as such is assayed in vivo as a bio-marker of oxidative stress (Stancliffe et al. 2011). These antioxidant results suggested that PEF could be used as a natural antioxidant to protect the human body from free radicals and retard the progress of many chronic diseases.

In the past few decades, a series of public health and hygiene incidents, such as the out break of AIV, have raised public concern about the deterioration of food safety and environmental hygiene standards. Moreover, as a result of the overuse of antibiotics, enhancement of viral resistance might lead to the appearance of a super virus. In this study, PEF was tested for inhibitory activity against AIV by means of a haemagglutination inhibition test. The results indicated that PEF could effectively interfere with viral adsorption and virus-cell binding. These results suggested that PEF might be used for the prophylaxis and treatment of diseases caused by AIV infection. Possible antiviral effects of PEF against human medical viruses such us influenza and herpes simplex need to be further examined and investigated.

In conclusion, all the results suggested that the protein-enriched fraction (PEF) from the larvae of housefly showed antiviral bioactivity against avian influenza virus H_9_N_2_, AcMNPV and BmNPV. It could modulate immune function in mice and possessed excellent DPPH radical and superoxide anion radical scavenging activities. PEF has the potential to be a natural antiviral, immunomodulatory and antioxidative agent to exploit in the future. These studies are very important in the utilization of housefly larvae and exploitation of their commercial value. Purification and functional evaluation of PEF will also be further investigated.

## Abbreviations

AcMNPV,: *Autographa californica* multicapsid nucleopolyhedrovirus;
**AIV**,: avian influenza virus H_9_N_2_;
**BmNPV**,: *Bombyx mori* nuclear polyhydrosis virus;
**DNFB**,: 2, 4-dinitrofluorobenzene;
**DPPH**,: 1,1-diphenyl-2- picrylhydrazyl;
**LPS**,: lipopolysaccharide;
**NKCA**,: natural killer cell activity;
**OB**,: occlusion bodies;
**PEF**,: protein-enriched fraction;
sf9 cell line,: *Spodoptera frugiperda* cell line 9
